# A proof-of-concept study on the effectiveness of botulinum toxin on spasticity plus syndrome in multiple sclerosis

**DOI:** 10.1007/s10072-025-08585-x

**Published:** 2026-01-03

**Authors:** Marcello Moccia, Chiara Clemente, Simone Braca, Antonio Carotenuto, Maria Petracca, Rosa Iodice, Roberta Lanzillo, Vincenzo Brescia Morra

**Affiliations:** 1 Department of Molecular Medicine and Medical Biotechnology, Federico II University of Naples, Naples, Italy; 2 Multiple Sclerosis Unit, Policlinico Federico II University Hospital, Naples, Italy; 3 Department of Neuroscience, Reproductive Science and Odontostomatology, Federico II University of Naples, Naples, Italy; 4 Department of Human Neuroscience, Sapienza University of Rome, Rome, Italy

**Keywords:** Botulinum toxin, Spasticity, Spasticity-plus syndrome, Multiple sclerosis

## Abstract

**Background:**

Botulinum toxin (BoNT) might improve spasticity-plus syndrome (SPS) in multiple sclerosis (MS) through peripheral inhibition of muscle contraction and central modulation of pain pathways, as hypothesized for migraine headaches. Hereby, we aim to explore changes in migraine headaches and SPS symptoms after BoNT treatment for MS-related spasticity.

**Methods:**

We recruited 9 people with MS who received BoNT injection due to spasticity and with significant impact due to migraine headaches (mean age 48.6 ± 6.4 years; 55.5% females; median EDSS 6.0). At the time of BoNT injection and after 1 and 3 months, patients filled in the Migraine Disability Assessment Test (MIDAS), the short form Headache Impact Test (HIT-6), the Migraine Specific Quality of Life Questionnaire (MSQ), the Beck Depression Inventory-II (BDI-II), the Fatigue Severity Scale (FSS), and the Pittsburgh Sleep Quality Index (PSQI).

**Results:**

On linear mixed-effect models, we observed significant improvements in MIDAS (Coeff=-2.61; 95%CI=-4.39, -0.83; *p* = 0.004), HIT-6 (Coeff=-1.89; 95%CI=-3.34, -4.45; *p* = 0.010), FSS (Coeff=-3.14; 95%CI=-5.62, -0.66; *p* = 0.013), and sleep efficiency (Coeff=-2.28; 95%CI=-4.17, -0.39; *p* = 0.018) and disturbance (Coeff=-0.18; 95%CI=-0.30, -0.06; *p* = 0.002), which were proportional to BoNT dosing.

**Conclusion:**

BoNT may represent a promising treatment for the management of SPS symptoms, possibly thanks to its peripheral and central effects.

## Introduction

The concept of spasticity-plus syndrome (SPS) was introduced in 2020 as a cluster of multiple sclerosis (MS) symptoms, including spasticity, spasms/cramps, pain, bladder dysfunction, sleep disorders, fatigue and tremor [1, 2]. Unlike traditional approaches that treat these symptoms separately, the SPS framework places them on the same level, suggesting a shared pathophysiological basis. The mechanisms underlying SPS are conduction block (linked to “negative” symptoms such as spasticity, fatigue, weakness, and urinary retention) and hyperexcitability of demyelinated fibers with ephaptic transmission (where damaged axons cause aberrant cross-stimulation, producing “positive” symptoms such as spasms, pain, allodynia, and urinary urgency) [1, 2].

Recognizing SPS as a distinct entity raises the possibility of targeting its common mechanisms with a single treatment, potentially improving multiple symptoms simultaneously, either directly or through secondary improvements in other symptoms [1–3]. Current literature often cites cannabinoid-based medicines, particularly nabiximols, as optimal symptomatic therapy for SPS [4]. Botulinum toxin (BoNT) is approved for the treatment of spasticity, but has the potential to improve other SPS-related symptoms [5, 6]. In particular, BoNT has established efficacy on pain syndromes, including migraine headaches [7]. In this specific case, the effect is not necessarily peripheral (i.e., related to the injection of BoNT in cranio-facial muscles), but could be equally due to diffusion into the central nervous system with modulation of the pain pathways [8–10]. As such, our hypothesis is that peripheral injection of BoNT for spasticity in MS can provide improvements in migraine headaches and, more in general, in components of SPS.

Hereby, we aim to explore changes in migraine headache and SPS symptoms during BoNT treatment for MS-related spasticity.

## Methods

### Study design and population

This is a proof-of-concept single center study. Patients referred to the BoNT clinic were consecutively recruited and screened for this study, with data collection from January 2024 to January 2025, and were then prospectively followed-up over 3 months following the injection. Ethics approval was obtained. All of the patients signed informed consent prior to the study and data processing authorization (GDPR 2016/679). The study was conducted in accordance with good clinical practice and the Declaration of Helsinki.

Inclusion criteria were: (1) diagnosis of MS; (2) clinically meaningful impact from migraine headaches (Headache Impact Test (HIT-6) questionnaire score ≥ 50) [11]; (3) clinical indication to BoNT injection due to MS-related spasticity; (4) consent to the study.

Exclusion criteria were: (1) concomitant diseases and/or treatments (at baseline or any changes during follow-up) possibly affecting study outcomes; (2) relapses, disability progression or MRI activity in the 6 months before the injection.

### Demographics and MS-related clinical variables

At the time of the BoNT injection (baseline), we collected demographic variables (age and sex) and the following MS-related clinical features: disease duration (years from reported disease onset to study inclusion, corresponding to BoNT injection), the Expanded Disability Status Scale (EDSS), disease course (relapsing or progressive), and the current disease-modifying treatment (DMT) [5, 12].

### Spasticity and BoNT injection variables

Spasticity was clinically defined as an increase in the velocity-dependent reflexes to phasic stretch, detected and measured at rest. The spasticity evaluation included a separate assessment of the tone in specific muscle groups (e.g., shoulder, elbow, wrist, fingers, hip, leg, knee, and ankle) by using the modified Ashworth score (MAS) (the minimum MAS score for the definition of spasticity was 1); a MAS score of 1 + was coded as 1.5 for results’ presentation. For each patient, at the time of the first BoNT injection, the highest MAS score among the injected muscles was used for results’ presentation [5, 12]. The concomitant spasticity treatments were also collected, as per the Italian consensus on the treatment of spasticity in MS [13].

We recorded formulation and dose of BoNT (abobotulinumtoxin A [Dysport^®^], incobotulinumtoxin A [Xeomin^®^], or onabotulinumtoxin A [Botox^®^]). The injection goals were classified using the World Health Organization (WHO) International Classification of Functioning, Disability, and Health (ICF) into the following categories: posturing/hygiene, mobility, pain, and daily assistance/functioning in daily living activities [5, 14]. Side effects were collected on follow-up assessments.

### Outcome measures

At baseline and after 1 and 3 months, patients filled in the following questionnaires:

- Migraine Disability Assessment Test (MIDAS), with higher scores indicating worse headache-related disability over a 3-month period [15];
- The short form Headache Impact Test (HIT-6), questionnaire with higher scores indicating worse impact of headaches [11];
- Migraine Specific Quality of Life Questionnaire (MSQ), with higher scores indicating worse quality of life due to migraines, and with subscores indicating domain-specific percent impairments (restrictive role function, preventive role function, emotional function) [16];
- Beck Depression Inventory-II (BDI-II), with higher scores indicating worse depressive symptoms [17];
- Fatigue Severity Scale (FSS), with higher scores indicating worse fatigue [18];
- Pittsburgh Sleep Quality Index (PSQI), with higher scores indicating worse sleep, and with subscores indicating domain specific impairments (subjective sleep quality, sleep latency, sleep duration, sleep efficiency, sleep disturbance, use of sleep medication, daytime dysfunction) [19].

### Statistical analyses

Results are presented as mean (and standard deviation), median (and range) or number (and percent), as appropriate.

To explore the variations in different outcomes over time within patients, we run linear mixed-effects regression models including, in turn, each outcome as dependent variable (MIDAS, HIT-6, MSQ and its subscores, BDI-II, FSS, PSQI and its subscores), and time as independent variable. Additional fixed-effect variables were age, sex, MS disease duration, EDSS, BoNT formulation and dosing (using unified dose units). We set a random subject intercept to account for the nested structure of the data (different measures over time within patients) [5, 12]. Coefficients (Coeff), 95% confidence intervals (95%CI) and p-vales were calculated. Results were considered statistically significant for *p* < 0.05.

Statistical analyses were performed with Stata 15.0.

## Results

During the recruitment period, we screened 122 people with MS who were referred to the BoNT clinic, and recruited 9 people with MS who received BoNT injection due to spasticity and with significant impact due to migraine headaches (HIT-6 ≥ 50). Demographic, BoNT injection and MS variables are reported in Table 1. No patient was lost to follow-up. During the follow-up, no patient had relapses, disability progression or MRI activity, and no side effects to BoNT were reported.

**Table 1:** Demographic, MS and BoNT injection variables

	*N* = 9
**Age**, years	48.6 ± 6.4
**Sex**, females	5 (55.5%)
**MS disease duration**, years	16.9 ± 6.8
**MS disease course**, progressive	8 (88.8%)
**EDSS**, median (range)	6.0 (2.5–7.5)
**Disease modifying treatment**	
*Cladribine*	1 (11.1%)
*Natalizumab*	1 (11.1%)
*Ocrelizumab*	6 (66.7%)
*Siponimod*	1 (11.1%)
**MAS**, median highest score (range)	2 (1.5–3)
**BoNT**	
**Formulation and dosing**	
*Onabotulinumtoxin A*	2 (22.2%) with 200 ± 0 units
*Abobotulinumtoxin A*	4 (44.4%) with 1500 ± 612.37 units
*Incobotulinumtoxin A*	3 (33.3%) with 233 ± 57.73 units
**Main injection goal**	
*Mobility*	3 (33.3%)
*Posturing/hygiene*	4 (44.4%)
*Pain*	2 (22.2%)
**Other medications for spasticity**, yes	6 (66.7%)

**Fig. 1:**
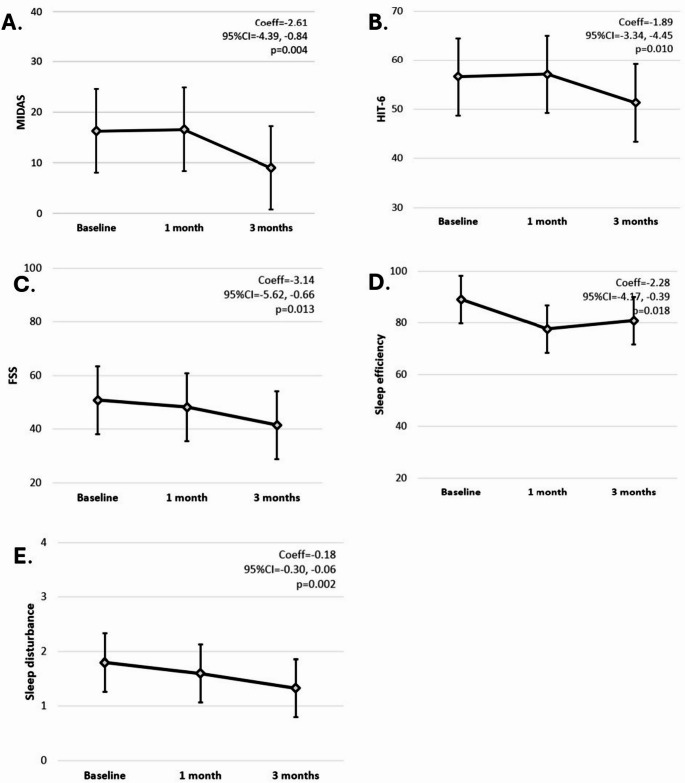
Changes in migraine headaches and spasticity plus syndrome. Profile plots show changes in MIDAS (**A**), HIT-6 (**B**), FSS (**C**), sleep efficiency (**D**) and sleep disturbance (**E**), between baseline, 1 month and 3 months, following BoNT injection. Coefficients (Coeff), 95% confidence intervals (95%CI), and p-values are reported from linear mixed-effect models

Over the 3-month follow-up, we observed significant improvements in HIT-6 (with 9 (100%) patients scoring ≥ 50 at baseline, 6 (75%) patients after 1 month, and 5 (55.5%) patients after 3 months), MIDAS, FSS, and sleep efficiency and disturbance (Table 2; Figs. 1). Improvements were dose-dependent (Table 2). We found no significant changes in MSQ (and subdomains), BDI-II, PSQI and subjective sleep quality, latency, duration, medications and daytime function (Table 2).

**Table 2:** Changes in migraine headaches and spasticity plus syndrome. Table shows absolute values at baseline, and after 1 month and 3 months from BoNT injection in MIDAS, HIT-6, MSQ (and subscores), BDI-II, FSS, and PSQI and subscores. Coefficients (Coeff), 95% confidence intervals (95%CI), and p-values are reported from linear mixed-effect models, including the effect of dosing for statistically significant models (*p* < 0.05)

	Baseline	1 month	3 months	Coeff	95%CI		*P*-value
**MIDAS**	16.3 ± 8.8	16.6 ± 10.5	9.0 ± 5.5	-2.61	-4.39	-0.83	*0.004*
				-0.04	-0.07	-0.01	*0.004*
**HIT-6**	56.6 ± 6.2	57.1 ± 8.3	51.3 ± 9.2	-1.89	-3.34	-4.45	*0.010*
				-0.02	-0.05	-0.01	*0.040*
**MSQ**	55.8 ± 16.4	55.7 ± 18.0	61.7 ± 19.6	1.97	-1.46	5.42	*0.261*
*Restrictive role function*	49.8 ± 17.9	49.6 ± 17.4	55.5 ± 23.3	1.92	-1.82	5.67	*0.315*
*Preventive role function*	57.7 ± 21.8	59.3 ± 19.8	67.7 ± 17.3	3.28	-0.55	7.12	*0.093*
*Emotional function*	67.4 ± 15.0	65.0 ± 19.7	68.1 ± 19.3	0.35	-3.59	4.31	*0.859*
**BDI-II**	11.5 ± 4.7	9.5 ± 8.4	10.8 ± 7.0	-0.16	-1.08	0.74	*0.719*
**FSS**	50.8 ± 10.1	48.2 ± 13.7	41.5 ± 14.1	-3.14	-5.62	-0.66	*0.013*
				-0.07	-0.11	-0.02	*0.002*
**PSQI**	5.0 ± 1.9	7.0 ± 2.7	4.8 ± 3.2	-0.08	-0.19	0.01	*0.093*
*Subjective sleep quality*	0.3 ± 1.0	0.1 ± 0.6	0.2 ± 0.7	0.05	-0.12	0.23	*0.560*
*Sleep latency*	0.8 ± 0.7	1.6 ± 0.7	1.0 ± 0.8	-0.01	-0.22	0.19	*0.904*
*Sleep duration*	0.5 ± 1.1	0.8 ± 1.1	0.7 ± 0.9	0.06	-0.08	0.20	*0.412*
*Sleep efficiency*	89.1 ± 6.8	77.7 ± 11.3	80.9 ± 9.5	-2.28	-4.17	-0.39	*0.018*
				-0.04	-0.06	-0.01	*0.033*
*Sleep disturbance*	1.8 ± 0.6	1.6 ± 0.5	1.33 ± 0.5	-0.18	-0.30	-0.06	*0.002*
				-0.02	-0.07	-0.01	*0.019*
*Use of sleep medication*	2 (11.1%)	2 (11.1%)	2 (11.1%)	n/a			
*Daytime dysfunction*	1.1 ± 0.6	1.2 ± 0.7	0.8 ± 0.6	-0.08	-0.19	0.01	*0.093*

## Discussion

BoNT treatment improves spasticity in MS [20] and, based on our proof-of-concept study, can offer dose-dependent improvements in migraine headaches, fatigue, and sleep quality, suggesting BoNT may act on shared neurophysiological pathways implicated in the broader constellation of MS and SPS symptoms. These findings lend support to the SPS framework, which posits that multiple MS-related symptoms may arise from overlapping mechanisms and respond to a unified therapeutic approach [1–3, 21].

We selected a population with impact from migraine headaches and indication to BoNT injection due to MS spasticity, and showed significant improvements in migraine headaches, as evidenced by reductions in both MIDAS and HIT-6 scores. This indicates that BoNT may alleviate headache-related disability and overall burden, domains traditionally considered outside the scope of spasticity management but likely within shared pathology mechanisms [1].

We also showed lower fatigue severity, fewer sleep disturbances, and improved sleep efficiency. These findings suggest a broader impact on patient well-being, potentially mediated by BoNT modulation of central and peripheral neural circuits involved in pain, muscle tone, and autonomic regulation [10, 22]. The temporal profile of symptom relief —emerging within days, peaking around one month, and diminishing by three months— closely mirrors BoNT known pharmacodynamics, thus making the case for a genuine therapeutic effect rather than random variation or placebo response.

The limited sample size, including only patients with progressive MS, constrains the generalizability of these findings. We limited the observation time to 3 months, so that sustained efficacy could not be explored. These limitations possibly apply also to the lack of significant effect on quality of life and depressive symptoms in our study. The observational and uncontrolled study design (i.e., lack of placebo group) do not allow any final conclusion on treatment effect, nor shed light on possible underlying mechanisms.

In conclusion, our hypothesis-generating study was based on the evidence that the effect of BoNT on migraine headaches goes beyond the injection of BoNT in the cranio-facial muscles, and can be related, at least in part, to the diffusion of BoNT in the central nervous system with modulation of pain pathways [8–10].The observed multi-symptom improvements carry important clinical implications. By conceptualizing SPS as an integrated clinical entity rather than a collection of isolated complaints, BoNT may represent a promising step toward more efficient and patient-centered symptom management in MS [23]. Notwithstanding study limitations, including the small sample size, the lack of a control group and the absence of long-term follow-up, the work opens up a stimulating perspective on an integrated approach to MS symptoms, highlighting BoNT as a possible option even for non-motor symptoms. Future research should prioritize large randomized-controlled trials to validate these preliminary findings, clarify underlying mechanisms, and identify patient subgroups most likely to benefit.

## Data Availability

Data are available upon reasonable request to the corresponding author.

## References

[1] Bruno A, Dolcetti E, Centonze D (2022) Theoretical and therapeutic implications of the Spasticity-Plus syndrome model in multiple sclerosis. Front Neurol 12:1–7. 10.3389/fneur.2021.802918 10.3389/fneur.2021.802918 PMC8859110 35197915

[2] Fernandez O, Costa-Frossard L, Martínez-Ginés ML et al (2021) Integrated management of multiple sclerosis spasticity and associated symptoms using the spasticity-Plus syndrome concept: results of a structured specialists’ discussion using the Workmat^®^ methodology. Front Neurol 12:1–11. 10.3389/fneur.2021.722801 10.3389/fneur.2021.722801 PMC8503561 34646229

[3] Bruno A, Annovazzi P, Clerico M et al (2024) Disease-Modifying symptomatic treatment (DMST) potential of cannabinoids in patients with multiple sclerosis. Curr Neuropharmacol 23:503–510. 10.2174/011570159x329058240820070701 10.2174/011570159X329058240820070701 PMC12163502 39279696

[4] Patti F, Chisari CG, Fernández Ó et al (2022) A real-world evidence study of nabiximols in multiple sclerosis patients with resistant spasticity: analysis in relation to the newly described ‘spasticity-plus syndrome’. Eur J Neurol 29:2744–2753. 10.1111/ene.15412 35590453 10.1111/ene.15412 PMC9539865

[5] Moccia M, Frau J, Carotenuto A et al (2020) Botulinum toxin for the management of spasticity in multiple sclerosis: the Italian botulinum toxin network study. Neurol Sci 41:2781–2792. 10.1007/s10072-020-04392-8 32281038 10.1007/s10072-020-04392-8

[6] Gold R, Oreja-Guevara C (2013) Advances in the management of multiple sclerosis spasticity: multiple sclerosis spasticity guidelines. Expert Rev Neurother 13:55–59. 10.1586/14737175.2013.865880 24289845 10.1586/14737175.2013.865880

[7] Corasaniti MT, Bagetta G, Nicotera P et al (2023) Safety of onabotulinumtoxin A in chronic migraine: A systematic review and Meta-Analysis of randomized clinical trials. Toxins (Basel) 15:1–20. 10.3390/toxins15050332 10.3390/toxins15050332 PMC10223125 37235366

[8] Ramachandran R, Yaksh TL (2014) Therapeutic use of botulinum toxin in migraine: mechanisms of action. Br J Pharmacol 171:4177–4192. 10.1111/bph.12763 24819339 10.1111/bph.12763 PMC4241086

[9] Turkel CC, Aurora S, Diener HC et al (2023) Treatment of chronic migraine with botox (onabotulinumtoxinA): Development, insights, and impact. Med (United States) 102:E32600. 10.1097/MD.0000000000032600 10.1097/MD.0000000000032600 PMC10374186 37499085

[10] Prudenzano MP (2024) Botulinum toxin and migraine: goals and perspectives. Toxins (Basel) 16:530. 10.3390/toxins16120530 39728788 10.3390/toxins16120530 PMC11679156

[11] Martin M, Blaisdell B, Kwong JW, Bjorner JB (2004) The Short-Form headache impact test (HIT-6) was psychometrically equivalent in nine languages. J Clin Epidemiol 57:1271–1278. 10.1016/j.jclinepi.2004.05.004 15617953 10.1016/j.jclinepi.2004.05.004

[12] Novarella F, Carotenuto A, Cipullo P et al (2022) Persistence with botulinum toxin treatment for spasticity symptoms in multiple sclerosis. Toxins (Basel) 14:1–8. 10.3390/toxins14110774 10.3390/toxins14110774 PMC9693315 36356024

[13] Comi G, Solari A, Leocani L et al (2019) Italian consensus on treatment of spasticity in multiple sclerosis. Eur J Neurol 27:445–453. 10.1111/ene.14110 31652369 10.1111/ene.14110

[14] World Health Organization (2020) ICF Browser. http://apps.who.int/classifications/icfbrowser/

[15] D’Amico D, Mosconi P, Genco S et al (2001) The migraine disability assessment (MIDAS) questionnaire: translation and reliability of the Italian version. Cephalalgia 21:947–952. 10.1046/J.0333-1024.2001.00277.X 11843865 10.1046/j.0333-1024.2001.00277.x

[16] Raggi A, Giovannetti AM, Schiavolin S et al (2014) Validating the Migraine-Specific quality of life questionnaire v2.1 (MSQ) in Italian inpatients with chronic migraine with a history of medication overuse. Qual Life Res 23:1273–1277. 10.1007/S11136-013-0556-9 24129671 10.1007/s11136-013-0556-9

[17] Solaro C, Trabucco E, Signori A et al (2016) Depressive symptoms correlate with disability and disease course in multiple sclerosis patients: an Italian multi-center study using the Beck depression inventory. PLoS ONE 11:1–9. 10.1371/journal.pone.0160261 10.1371/journal.pone.0160261 PMC5025048 27632167

[18] Ottonello M, Pellicciari L, Giordano A, Foti C (2016) Rasch analysis of the fatigue severity scale in Italian subjects with multiple sclerosis. J Rehabil Med 48:597–603. 10.2340/16501977-2116 27344968 10.2340/16501977-2116

[19] Curcio G, Tempesta D, Scarlata S et al (2013) Validity of the Italian version of the Pittsburgh sleep quality index (PSQI). Neurol Sci 34:511–519. 10.1007/s10072-012-1085-y 22526760 10.1007/s10072-012-1085-y

[20] Schramm A, Ndayisaba J, auf dem Brinke M et al (2014) Spasticity treatment with onabotulinumtoxin A : data from a prospective German real-life patient registry. J Neural Transm 121:521–530. 10.1007/s00702-013-1145-3 24407377 10.1007/s00702-013-1145-3

[21] Goicochea Briceño H, Higueras Y, Ruiz Pérez I et al (2024) Spasticity-Plus syndrome in multiple sclerosis patients in a tertiary hospital in Spain. Front Neurol 15:1–8. 10.3389/fneur.2024.1360032 10.3389/fneur.2024.1360032 PMC10926473 38469589

[22] Raciti L, Raciti G, Militi D et al (2022) Chronic migraine: A narrative review on the use of botulinum toxin with clinical indications and future directions. J Integr Neurosci 21. 10.31083/j.jin2105141 10.31083/j.jin2105141 36137961

[23] Fernández Fernández Ó, Costa-Frossard L, Martínez Ginés ML et al (2024) Practical tool to identify Spasticity-Plus syndrome amongst patients with multiple sclerosis. Algorithm development based on a conjoint analysis. Front Neurol 15:1–10. 10.3389/fneur.2024.1371644 10.3389/fneur.2024.1371644 PMC11066270 38708001

